# Spiking Neural Network for Fourier Transform and Object Detection for Automotive Radar

**DOI:** 10.3389/fnbot.2021.688344

**Published:** 2021-06-07

**Authors:** Javier López-Randulfe, Tobias Duswald, Zhenshan Bing, Alois Knoll

**Affiliations:** Department of Informatics, Technical University of Munich, Munich, Germany

**Keywords:** spiking neural network, FMCW radar, Fourier tranform, constant false-alarm rate, autonomous driving

## Abstract

The development of advanced autonomous driving applications is hindered by the complex temporal structure of sensory data, as well as by the limited computational and energy resources of their on-board systems. Currently, neuromorphic engineering is a rapidly growing field that aims to design information processing systems similar to the human brain by leveraging novel algorithms based on spiking neural networks (SNNs). These systems are well-suited to recognize temporal patterns in data while maintaining a low energy consumption and offering highly parallel architectures for fast computation. However, the lack of effective algorithms for SNNs impedes their wide usage in mobile robot applications. This paper addresses the problem of radar signal processing by introducing a novel SNN that substitutes the discrete Fourier transform and constant false-alarm rate algorithm for raw radar data, where the weights and architecture of the SNN are derived from the original algorithms. We demonstrate that our proposed SNN can achieve competitive results compared to that of the original algorithms in simulated driving scenarios while retaining its spike-based nature.

## 1. Introduction

Autonomous driving is a billion-dollar business with high demand for efficient computing systems. This introduces a limitation for highly automated vehicles, where the systems that process sensor data can drain more than 10% of the power stored for driving (Lin et al., 2018). Radar sensors are a fundamental component of most autonomous vehicles. Their low price and robustness against bad weather and lighting conditions make them great companions for lidar and vision cameras (Hasch et al., 2012; Winner et al., 2014; Patole et al., 2017). Whereas, the algorithms in this field are typically implemented on graphical processing units (GPUs), field-programmable gate arrays (FPGAs), or application-specific integrated circuits (ASICs) (Lin et al., 2018), neuromorphic hardware (NHW) offers an efficient alternative environment (Furber et al., 2014; Davies et al., 2018; Sangwan and Hersam, 2020). It provides a low-energy-footprint platform for a new generation of neural networks called spiking neural networks (SNNs), which reduce the high energy consumption of the popular artificial neural networks (ANNs) (Maass and Schmitt, 1999; Bouvier et al., 2019; Strubell et al., 2019).

SNN-based applications for sensor signal processing comprise a novel area within autonomous driving and have been applied to traditional vision cameras for tracking applications (Cao et al., 2015; Piekniewski et al., 2016); raw temporal pulses of lidar sensors for object detection (Wang et al., 2021); dynamic vision sensors for tasks including lane keeping (Bing et al., 2020), feature extraction and motion perception (Paredes-Vallés et al., 2019), and collision avoidance (Salvatore et al., 2020); as well as remote sensing images for object detection (Liu et al., 2018). Likewise, bio-inspired neural networks have also been applied to control problems in mobile applications, including rotation control for unmanned aerial vehicles (Stagsted et al., 2020); and tracking control (Khan et al., 2021) and obstacle avoidance (Khan et al., 2020) for smart-home manipulators.

Current trends in signal processing include optimization of the energy consumption, memory resources, and computational speed of the Fourier transform (FT) (Gilbert et al., 2014). In the case of radar processing, the FT converts the raw data from the sensor into a range-Doppler map, followed by an object-detection algorithm that separates the target values from noise (Patole et al., 2017). Later stages extract high-level information, such as a list of labeled objects or target trajectories. A few recent works have explored the application of SNNs to decomposing a time signal into a frequency spectrum, e.g., by applying sequential spiking band-pass filters to audio signals (Jiménez-Fernández et al., 2016) or using neurons that spike at specific input frequencies (Auge and Mueller, 2020). The former offers an efficient bio-inspired solution, but its applications are limited to extracting a small set of frequency components. The latter provides a simple and elegant solution, but fails to provide an accurate measurement of the frequency-components angles, which are crucial for computing a 2D FT.

In this letter, we present a novel SNN that is able to effectively replace the discrete FT (DFT) and constant false-alarm rate (CFAR) algorithms (Rohling, 1983). Its weights are fixed, as they are mathematically derived from the equations defining the two algorithms, leading to equivalent results in simulated driving scenarios. Therefore, the network does not require learning to adapt its weights. Modern radar applications use the fast FT (FFT), which consists of a recursive decomposition of the problem into smaller DFTs over subsets of the original data (Frigo and Johnson, 2005), i.e., the DFT is a generalization of the FFT. The proposed network is a theoretical approximation of the DFT, and further work can explore network topologies with smaller layers that mimic the desired FFT structure. The quantitative results show high similarity between the outputs of the proposed SNN and the original algorithms for both one and two dimensions. In combination with NHW, this work could provide an efficient alternative for processing radar data while maintaining analogous performance. Designing SNNs that can process sensor data is a crucial step for obtaining full neuromorphic sensor processing pipelines and, together with NHW, bring a new generation of signal-processing solutions with higher energy efficiencies and shorter latency response-times. Furthermore, finding SNN equivalents for all radar-processing stages is paramount, as hybrid pipelines introduce additional complexity through communication and spike conversion when data flows between the NHW and traditional hardware.

## 2. Spiking Neural Network

**Figure 1:**
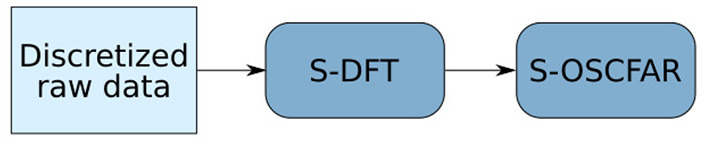
Diagram of the implemented pipeline. The first block implements a spiking DFT on raw discretized FMCW radar data, and the second block implements the spiking OS-CFAR.

In this section, we explain an SNN that substitutes the 2D DFT (S-DFT) and object detection (S-OSCFAR) on raw frequency-modulated continuous-wave (FMCW) radar data. To do so, the network is split into two smaller networks that process each of the steps (see Figure 1).

### 2.1. Fourier Transform

The 2D DFT is a linear transformation that can be represented by two successive matrix multiplications. Hence, each of the dimensions of the 2D DFT can be represented by a one-layer neural network with a linear activation function.

**Figure 2:**
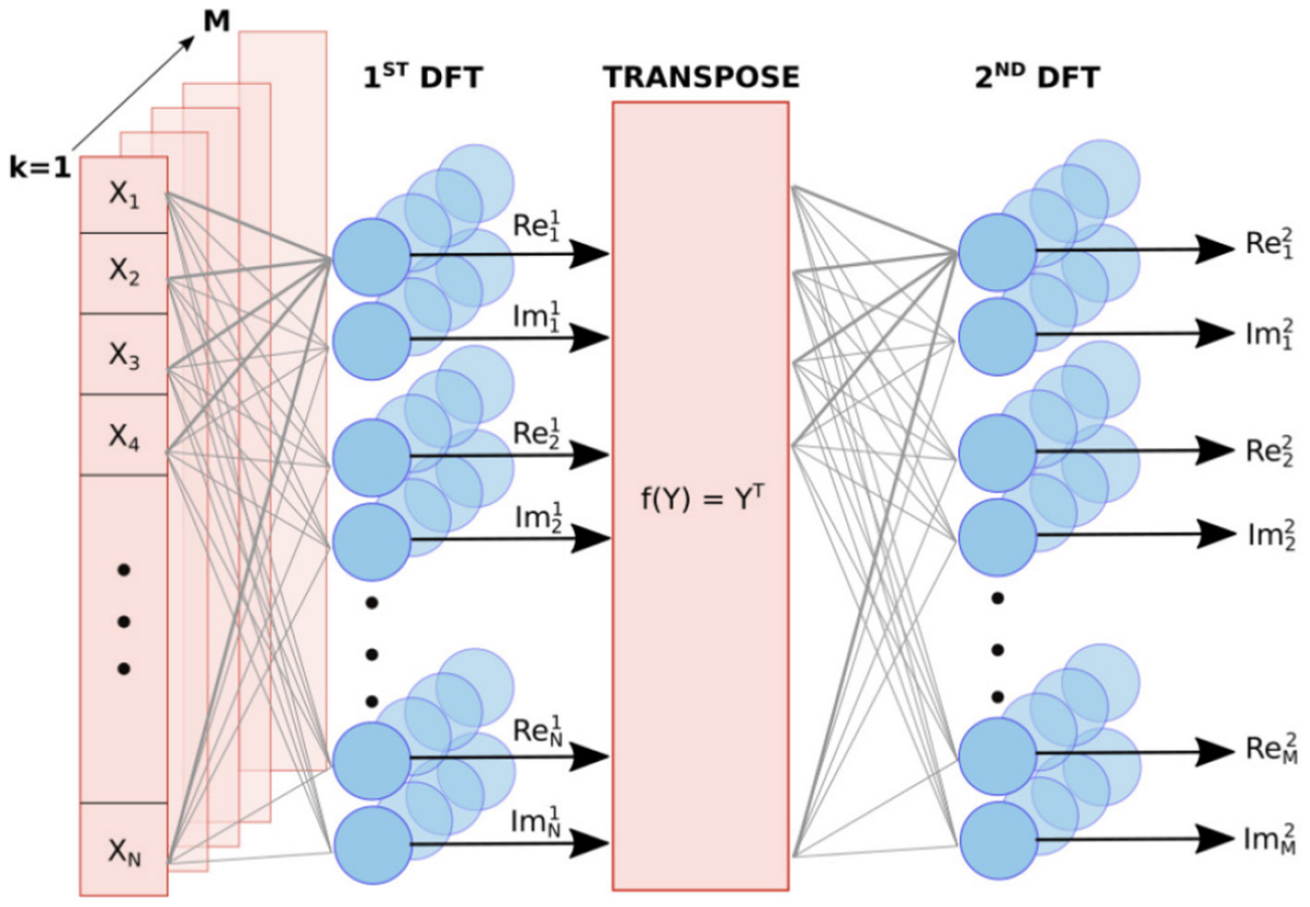
Diagram of the S-DFT. The first layer implements one DFT per chirp to obtain the range-dependent frequency bins, and the second layer performs a DFT along the different chirps to obtain the Doppler frequency components. A transpose operation is implemented between these steps, as each layer is applied along a different dimension.

We have implemented the S-DFT with a two-layer network, where the first layer provides information about the target ranges and the second extracts their velocities. The input data has dimension *N* × *M*, where *N* is the total number of samples per chirp, and *M* is the number of chirps in a radar frame. Owing to the complex nature of the DFT, both the real and imaginary values must be computed. Therefore, each layer contains 2*N* × *M* neurons (Figure 2).

The weights of the network have been calculated based on the trigonometric equation of the DFT:

(1) $$\begin{array}{l} Y_{k}=\overset{L-1}{\underset{l=0}{\sum }}X_{l}\left[cos\left(\frac{2\pi }{L}kl\right)-i\cdot sin\left(\frac{2\pi }{L}kl\right)\right]. \end{array}$$

Both the input vector *X* and output vector *Y* are formed by *L* values; hence, *k* and *l* take values between 0 and *L* − 1. *L* takes the value of *N* for the first layer and *M* for the second. We rewrite Equation (1) as

(2) $$\begin{array}{l} Y=W_{Re}X+i\cdot W_{Im}X, \end{array}$$

where *Y* is the result of the transform, *X* is the input vector, and *W*_*Re*_ and *W*_*Im*_ are the real and imaginary coefficients, respectively. From Equation (1), the individual weight coefficients can be calculated using the following equations:

(3) $$\begin{array}{l} w_{{Re}_{kl}}=cos\left(\frac{2\pi }{L}kl\right), \end{array}$$

(4) $$\begin{array}{l} w_{{Im}_{kl}}=-sin\left(\frac{2\pi }{L}kl\right). \end{array}$$

The first layer is replicated in parallel *M* times using the same weights. The output consists of the frequency spectrum of the input chirps, formed each by *2N* values.

The second layer applies the same algorithm to the output of the first layer, *Y*^(1)^. The main difference is that the input in this case is formed by complex values, so the real and imaginary parts obtained from Equation (2) for the first layer are fed into the equation for the second layer. Therefore, Equation (2) can be generalized for the second layer by using the following algebraic system:

(5) $$\begin{array}{l} \left[\begin{array}{c} Re\left(Y^{\left(N\right)}\right)\\ Im\left(Y^{\left(N\right)}\right) \end{array}\right]=[\begin{array}{cc} W_{Re} & W_{Im}\\ -W_{Im} & W_{Re} \end{array}]\;\left[\begin{array}{c} Re{\left(Y^{\left(N-1\right)}\right)}^{T}\\ Im{\left(Y^{\left(N-1\right)}\right)}^{T} \end{array}\right]. \end{array}$$

For this layer, *W*_*Re*_ and *W*_*Im*_ are *M* × *M* matrices.

With appropriate weights, a two-layer feed-forward network with linear activation can represent Equations (2) and (5). A rate-based SNN can approximate a neural network based on rectified linear unit (ReLU) functions (Rueckauer et al., 2017). For one layer, such a network transforms its input *a* ∈ ℝ^*n*^ as

(6) $$\begin{array}{l} z_{j}=\text{ReLU}\left(\underset{i}{\sum }W_{ji}a_{i}\right), \end{array}$$

where *W* ∈ ℝ^*m*×*n*^ is the weight matrix and *z* ∈ ℝ^*m*^ is the result. In the rate-based approximation, the input is represented by *n* neurons whose spike frequencies are proportional to the components of *a*. Assuming that we simulate the SNN for *k* timesteps, we denote the spike train of the input neuron *i* as a binary vector Θ^(*i*)^ ∈ {0, 1}^*k*^, where 1 in indicates a spike and Θ^(*i*)^ is computed from *a*_*i*_. Rueckauer et al. (2017) showed that *z* can be approximated by the spike frequency of *m* output neurons. The membrane potential *V*_*j*_ fully describes the state of the output neuron *j*. At a certain timestep 1 < *t* < *k*, it is computed according to

(7) $$\begin{array}{l} V_{j}^{t}=V_{j}^{t-1}+V_{thr}\underset{i}{\sum }W_{ji}\Theta_{t}^{\left(i\right)}, \end{array}$$

where *V*_*thr*_ is a model parameter. We compute the output spike train with a threshold behavior. Whenever $V_{j}^{t}>V_{thr}$, the output neuron *j* spikes and $V_{j}^{t}$ is reduced by *V*_*thr*_. The spike train defines the spike frequency, and we obtain an approximation to *y*_*j*_. For deeper networks, the neuron model governs all hidden layers as well as the output layer (Rueckauer et al., 2017).

Latest conversion approaches are based on ReLU networks. However, a single ReLU function does not span over the entire real spectrum, so the S-DFT implements the activation by combining two ReLU functions with opposite signs in their weights. To still use the existing conversion theory, we rewrite a regular matrix multiplication as a sum of two ReLU layers

(8) $$\begin{array}{l} \begin{array}{c} \underset{i}{\sum }W_{ji}a_{i}=\text{ReLU}\left(\underset{i}{\sum }W_{ji}a_{i}\right)-\text{ReLU}\left(\underset{i}{\sum }\left(-W_{ji}\right)a_{i}\right) \end{array}, \end{array}$$

which leads to two convertible sub-networks with weights of opposite signs. This logic is applied to Equations (1) and (5) in order to obtain a convertible network and ultimately an SNN for the DFT.

### 2.2. OS-CFAR

CFAR algorithms differentiate signals from noise. In the context of radar processing, the ordered-statistics (OS) CFAR is a very prominent candidate to detect targets in range-Doppler maps (Rohling, 1983).

**Figure 3:**
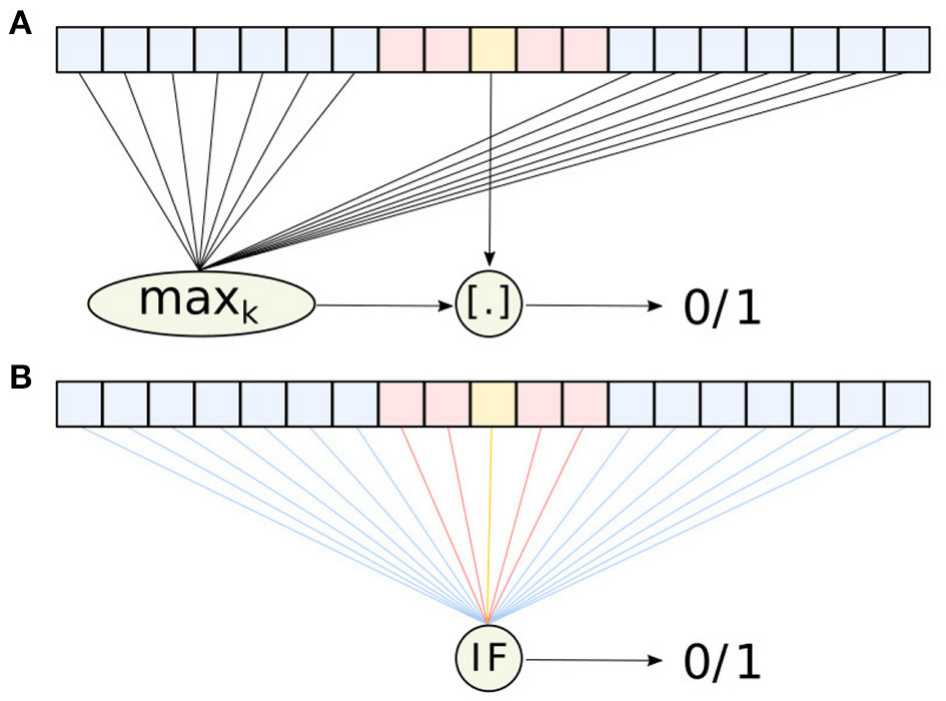
Diagrams representing the functionality of **(A)** the traditional OS-CFAR algorithm, and **(B)** the spiking OS-CFAR algorithm described in section 2.2. The diagrams show the *N* neighbor cells, *G* guarding cells, and the value under consideration *x*_*c*_ in blue, red, and yellow, respectively. The different connection colors indicate the weights: −1 (blue), 0 (red), and 1 + (*k* − 1) (yellow).

It operates on a 1D array *x* containing *N* + *G* + 1 values, where *N* is the number of neighbor cells, *G* is the number of guarding cells, and the remaining value is the one to be classified (see Figure 3). Let us denote the latter as *x*_*c*_ and the set of all neighbor cells as N. The OS-CFAR algorithm compares the *kth* largest value of N against α*x*_*c*_, where 0 < α < 1 is a scale factor. If α*x*_*c*_ is larger, it is classified as a signal; otherwise, it is considered noise. This can be expressed as

(9) $$\begin{array}{l} \text{OS-CFAR}\left(x\right)=\left[\alpha x_{c}>max_{k}\left\{y|y\in N\right\}\right], \end{array}$$

where *max*_*k*_ selects the *kth* largest element and [.] denotes the Iverson bracket^1^.

The OS-CFAR algorithm can be replaced by an SNN consisting of a single integrate-and-fire (IF) neuron, see Equation (7). We refer the reader to chapter 1.3 of Gerstner et al. (2014) for more details about the neuron model. If the IF neuron receives a spike from a pre-synaptic neuron connected with weight *w*, the membrane potential *v* instantaneously increases to the new value, *v* + *w*. The information of the real-valued vector *x* ∈ ℝ^*q*^ is encoded in the precise spike time of a set of *q* pre-synaptic neurons. For a given value *x*_*i*_, the associated pre-synaptic neuron *i* spikes at

(10) $$\begin{array}{l} t\left(x_{i}\right)=-\left(t_{max}-t_{min}\right)\frac{x_{i}-x_{min}}{x_{max}-x_{min}}+t_{max}, \end{array}$$

where *x*_*min*_ < *x*_*i*_ < *x*_*max*_ ∀*i*, and *t*_*min*_ and *t*_*max*_ define the earliest and latest spike time, respectively. Equation (10) is a linear transformation that maps larger values to earlier spike times.

The defined encoding allows us to rewrite the CFAR problem. Instead of finding the *kth* largest value and comparing it against α*x*_*c*_, we evaluate whether fewer than *k* spikes arrive before *t*(α*x*_*c*_). Therefore, we need to choose the parameters of the IF neuron appropriately. Among many different choices, we propose defining the initial membrane potential, firing threshold, neighbor weights, guarding weights, and weight for the value under consideration as *v*_0_ = 0, *v*_*th*_ = 1, $w_{N}=-1$, $w_{G}=0$, and *w*_*c*_ = 1 + (*k* − 1), respectively. With these parameters, the neuron spikes if and only if fewer than *k* spikes arrived before *t*(α*x*_*c*_). The neuron spikes in the same manner in which the OS-CFAR computes 0 or 1; thus, in that sense, both methods are mathematically equivalent. To guarantee that the classic algorithm and its spiking counterpart detect the same peaks, the time step Δ*t* must comply with Δ*t* < |*t*_*k*−1_ − *t*(α*x*_*c*_)|; i.e., the (*k* − 1)*th* spike must arrive at least one time step before the reference spike.

The method can be generalized to the 2D case by defining the neighbor and guarding cells on a 2D array, as done for the traditional OS-CFAR algorithm. The interested reader may find more information in Kronauge and Rohling (2013). Although the weights and spike times remain unchanged, the algorithmic parameters *k* and α need to be adapted to the new problem to obtain meaningful results (Rohling, 1983).

## 3. Experiment Results

The data used in the experiments has been gathered using an automotive radar simulator. This tool simulates a 77 GHz radar with a user-specified number of targets in the sensed scene. The generated data also includes several sources of noise typically present in real radar data, e.g., analog front-end noise, ADC noise and saturation, phase noise, and thermal noise. The generated data frames are formed by 128 chirps and 1,024 samples per chirp. The bandwidth and duration of each chirp are 275 MHz, and 54 μ*s*, respectively.

The simulated sensor is a long-distance radar (range up to 300 m), and the scene is populated with three targets at ranges *R* = {5, 9, 100} *m*. The velocities of the targets relative to the radar are *V* = {0, 2, 14} *m*/*s*, and the radar cross section (RCS) σ = {0, 5, 40} *dBsm*. The first two objects represent two pedestrians, whereas the last target represents a vehicle. These values are based on the typical velocities of such targets, and the RCS values are obtained from previous studies (Kamel et al., 2017; Deep et al., 2020). The simulation also includes 20-mm sensor packaging and the 1-cm car bumper, with RCS σ_*P*_ = −40 *dBsm*, and σ_*B*_ = −23 *dBsm*, respectively.

The proposed algorithm was implemented in Python, and the code is open-source^2^.

### 3.1. One-Dimensional DFT and CFAR

In the first experiment, we applied the S-DFT and S-OSCFAR to a single chirp of the simulated scene. The 1D S-DFT was simulated with 1,000 time steps of size Δ*t* = 0.01ms, i.e., for a total time of 10ms. The input values are converted to spike trains with regular spike time intervals Δ*T* ∈ [0.2, 10.0]ms. To encode the S-OSCFAR values, we set the parameters *t*_*min*_ and *t*_*max*_ to 0 and 50ms, respectively. This sub-network was also simulated with a temporal resolution Δ*t* = 0.01ms. Both the classical and spiking CFAR algorithms used *G* = 12 guarding cells, *N* = 30 neighbor cells, a scale factor α = 0.2, and compared against the *kth* = 6*th* largest value.

**Figure 4:**
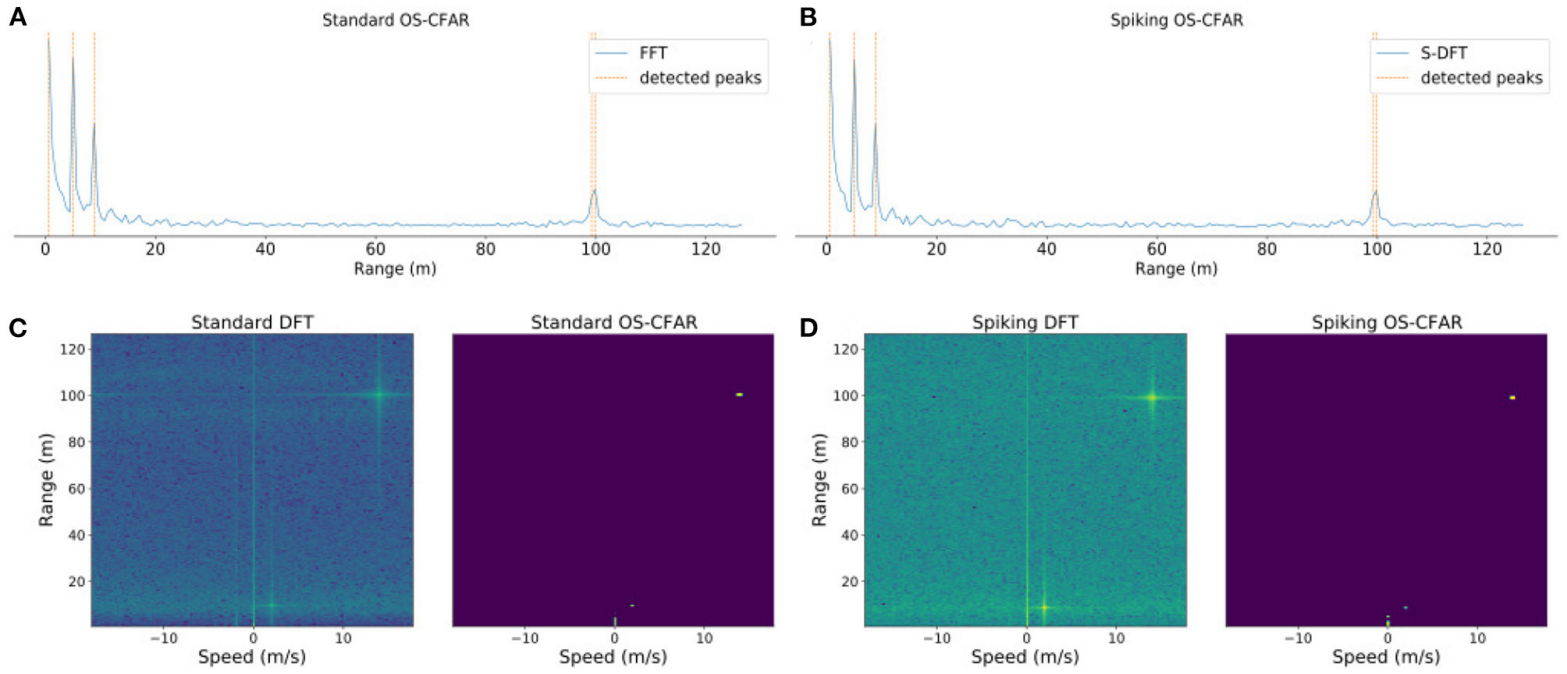
Results of the experiments on a simulation with three targets and a car bumper next to the sensor for **(A)** the 1D standard DFT and OS-CFAR algorithms, **(B)** the 1D S-DFT and S-OSCFAR, **(C)** the 2D standard DFT and OS-CFAR algorithms, and **(D)** the 2D S-DFT and S-OSCFAR. The sensor range is up to 300 m, but for visualization purposes the range dimension has been cropped until 125 m.

Figure 4A shows the results of the original algorithms, and Figure 4B shows the results of the proposed algorithm for a single dimension, i.e., range. The performance of both the standard and spiking versions are comparable, and they were able to detect the three objects present in the scene.

For evaluating the performance of the S-DFT, we measured the root mean square error (RMSE) between the DFT and S-DFT, which yielded RMSE = 0.0056. This measure was taken after normalizing the output values between 0 and 1. This small error is generated during the encoding process, as the S-DFT is a mathematical approximation of the original DFT.

### 3.2. Two-Dimensional DFT and CFAR

In the second experiment, we applied the S-DFT and S-OSCFAR to the complete radar frame of the simulated scene. The 2D S-DFT simulation parameters were identical to the 1D case, except for the total simulation time, which was increased to 50ms. Furthermore, the encoding parameters for the S-OSCFAR algorithm and the temporal resolution were identical to the 1D experiment. The CFAR parameters were set to *G* = 48 guarding cells, *N* = 176 neighbor cells, a scale factor α = 0.2, and compared against the *kth* = 9*th* largest value.

Figure 4C shows the results for the standard DFT and OS-CFAR, and Figure 4D shows the results for the spiking versions. As in the 1D experiment, the traditional and the spiking approaches were able to detect the three targets.

The error between the traditional algorithm and the S-DFT resulted in an RMSE of 0.0060, which is slightly larger than in the 1D case. As the number of layers in the network increases, encoding errors accumulate and the results increasingly differ from the desired goal (Rueckauer et al., 2017). However, this error is small, and the S-DFT offers very similar output to that of the standard DFT. Moreover, the small deviations did not affect the overall detection performance.

## 4. Conclusion

In this work, we presented an SNN that approximates the first stages of a typical automotive radar pipeline i.e., the Fourier transform and object detection. In contrast to the majority of ANN and SNN applications, our network is mathematically derived from the original algorithms and is not based on learning, as the DFT and CFAR are efficient and fast methods that can be translated into a neural network. Thus, we avoid the difficulties of network verification arising from high-dimensional optimization algorithms.

The implementation of every step of the radar signal processing with SNNs is fundamental in order to unfold the full potential of SNNs and NHW. For instance, the input and output of the proposed method are binary temporal events. In the present work, this data structure is inferred with encoding and decoding techniques. However, they introduce additional overhead and consequently penalize the energy footprint of the method. Instead of generating spikes after the analog-to-digital converter, future work should focus on designing *ad-hoc* electronics for generating spike-trains directly from the voltage signal, taking inspiration from the most recent advances in neuromorphic sensors. Future work should also focus on encoding the information using temporal coding, due to the efficiency gains that it provides, as well as extending the SNN to higher-level processing stages e.g., classification, tracking, or semantic segmentation. Furthermore, it is necessary to implement the developed algorithms in NHW and conduct benchmarking experiments that compare them with traditional methods. An exhaustive assessment that evaluate performance parameters (e.g., latency, energy, and memory usage) is paramount in order to determine its real potential for automotive applications.

The application of SNNs has typically been limited to computer vision datasets or *ad-hoc* neuromorphic sensors, and, to our knowledge, this is the first implementation of an SNN to the processing pipeline of automotive radar. We anticipate a rise in the application of SNNs for radar processing in upcoming years, due to increasing interest in efficient processing chains for autonomous driving applications and the major role that radar sensors play in these systems.

## Data Availability Statement

The data for conducting the experiments is available here: https://github.com/KI-ASIC-TUM/spiking-dft-cfar.

## Author Contributions

JL-R designed and implemented the S-DFT. TD designed and implemented the S-OSCFAR. The manuscript was written by JL-R and TD, with support from ZB. AK supervised the project and provided the funding. All authors contributed to the article and approved the submitted version.

## Conflict of Interest

The authors declare that the research was conducted in the absence of any commercial or financial relationships that could be construed as a potential conflict of interest.

## Footnotes

^1^The Iverson bracket [*C*] yields 1 if the condition *C* is true and 0 if false.

^2^https://github.com/KI-ASIC-TUM/spiking-dft-cfar
